# Contractility analysis of human engineered 3D heart tissues by an automatic tracking technique using a standalone application

**DOI:** 10.1371/journal.pone.0266834

**Published:** 2022-04-14

**Authors:** José M. Rivera-Arbeláez, Carla Cofiño-Fabres, Verena Schwach, Tom Boonen, Simone A. ten Den, Kim Vermeul, Albert van den Berg, Loes I. Segerink, Marcelo C. Ribeiro, Robert Passier

**Affiliations:** 1 Applied Stem Cell Technologies, TechMed Centre, University of Twente, Enschede, The Netherlands; 2 BIOS Lab-on-a-Chip Group, MESA+ Institute for Nanotechnology, Technical Medical Centre, Max Planck Center for Complex Fluid Dynamics, University of Twente, Enschede, The Netherlands; 3 River BioMedics, Enschede, The Netherlands; 4 Department Anatomy and Embryology, Leiden University Medical Centre, Leiden, The Netherlands; Purdue University, UNITED STATES

## Abstract

The use of Engineered Heart Tissues (EHT) as *in vitro* model for disease modeling and drug screening has increased, as they provide important insight into the genetic mechanisms, cardiac toxicity or drug responses. Consequently, this has highlighted the need for a standardized, unbiased, robust and automatic way to analyze hallmark physiological features of EHTs. In this study we described and validated a standalone application to analyze physiological features of EHTs in an automatic, robust, and unbiased way, using low computational time. The standalone application “*EHT Analysis*” contains two analysis modes (automatic and manual) to analyzes the contractile properties and the contraction kinetics of EHTs from high speed bright field videos. As output data, the graphs of displacement, contraction force and contraction kinetics per file will be generated together with the raw data. Additionally, it also generates a summary file containing all the data from the analyzed files, which facilitates and speeds up the post analysis. From our study we highlight the importance of analyzing the axial stress which is the force per surface area (μN/mm^2^). This allows to have a readout overtime of tissue compaction, axial stress and leave the option to calculate at the end point of an experiment the physiological cross-section area (PSCA). We demonstrated the utility of this tool by analyzing contractile properties and compaction over time of EHTs made out of a double reporter human pluripotent stem cell (hPSC) line (NKX2.5^EGFP/+-^COUP-TFII^mCherry/+^) and different ratios of human adult cardiac fibroblasts (HCF). Our standalone application “EHT Analysis” can be applied for different studies where the physiological features of EHTs needs to be analyzed under the effect of a drug compound or in a disease model.

## Introduction

Cardiovascular diseases (CVD) have been the leading cause of death and disability worldwide and are one of the costliest chronic diseases [1]. CVD costs are expected to increase substantially as the life expectancy increases. Innovative approaches are needed to bring new treatments to the market more quickly and cost-effectively [2–4]. For this, cardiac models can be used, such as 2D *in vitro* models or animal models. The former is often too simple and do not accurately represent the efficacy and safety of drug compounds in humans. The responses seen in animal models cannot always be directly related to the human situation. Therfore, these two cardiac models face both short comings, which contributed to a high compounds failure rate in clinical trials [5–7]. Instead, the use of *in vitro* 3D cardiac models, such as engineered heart tissues (EHT) have shown the advantage to mimic *in vivo* organization, functionality and cell-cell interaction, essential to resemble the human heart to study the pharmacodynamics and pharmacokinetics during preclinical studies of drug development.

The contraction force is the main feature of the human heart that allows pumping blood through the vasculature. The physiological performance of this contraction is crucial to assess the heart function following treatment of drug compounds or when evaluating a disease phenotype [8–10]. The EHTs have shown to be a gold standard 3D *in vitro* model for mimicking the contractility of the cardiac tissue by using human pluripotent stem cell-derived cardiomyocytes (hPSC-CMs) combined with environmental stimulation (mechanical and electrical) [11–15]. Cardiomyocytes (CMs) have the ability to organize into 3D myocardial structures when suspended in an extracellular matrix (ECM) surrounding anchor points. These anchor points create a mechanical restriction on the CMs that induce an enhancement of the structural, metabolic and physiological maturity of hPSC-CMs [16–18]. These EHT models are beeing used by pharmaceutical companies and academia for drug discovery and disease modeling, as they provide important insights into the genetic mechanisms, cardiac toxicity or drug responses [19, 20]. With the increased use of EHT models, an unbiased, robust and automatic way to analyze the contractile properties of the 3D cardiac tissues is crucial. Here we developed a software tool that fullfills these criteria which enables analysis of the contractile properties and contraction kinetics of EHTs. As output, an excel file containing the data, graphs of displacement, contraction force and contraction kinetics per file are generated. Additionally, a summary file with all the data from the analyzed files is made, which facilitates and speeds up the post analysis. We demonstrated the utility of this tool by analyzing contractile properties and compaction over time of hPSC-EHTs with different ratios of human adult cardiac fibroblast (HCF).

## Materials & methods

### HPSC culture and generation of hPSC cardiomyocytes

**Fig 1:**
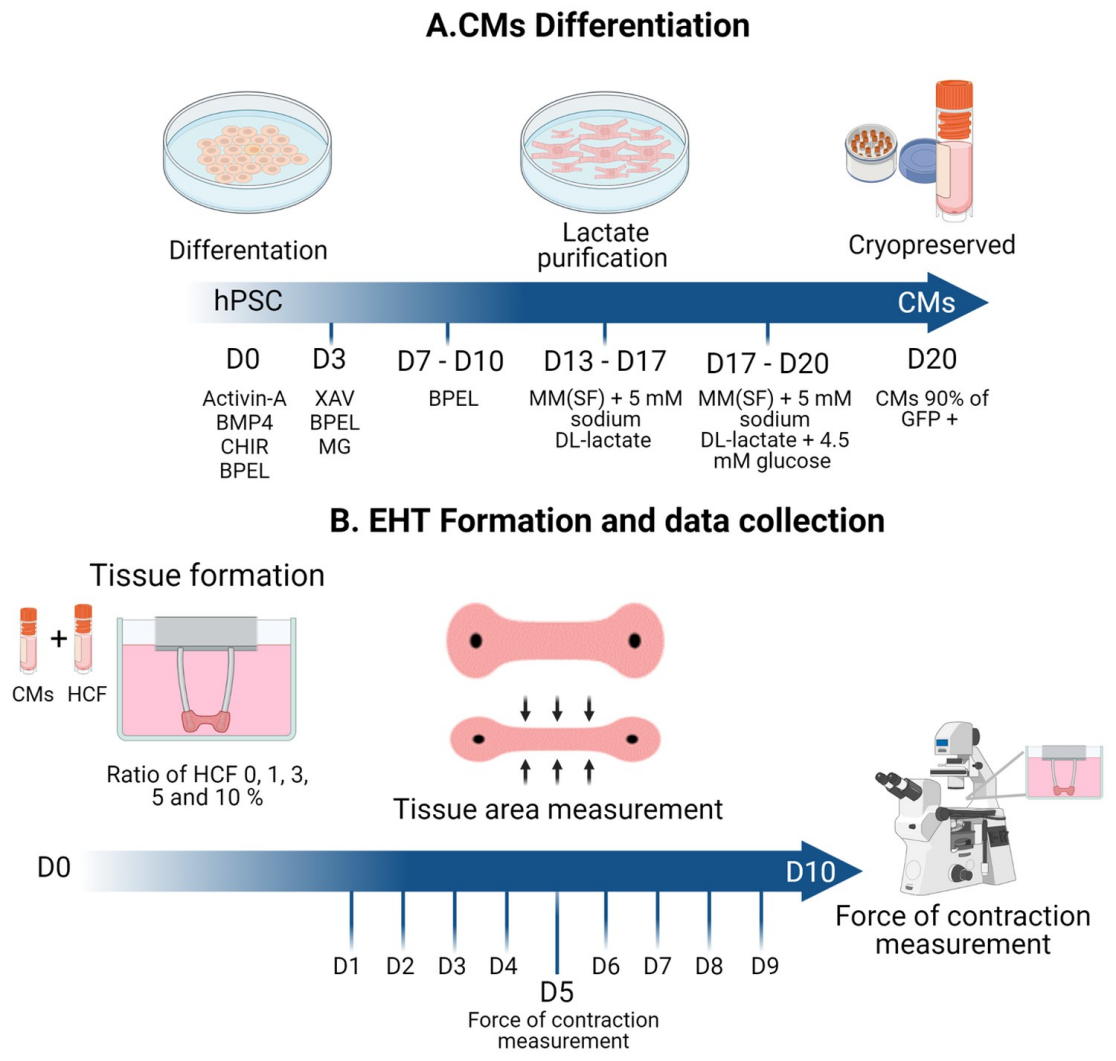
Experimental flow chart. (A) CMs differentiation steps from day 0 (D0), lactate purification at day 13 (D13) and cryopreservation of CMs at day 20 (D20). (B) EHT formation from frozen CMs and different ratios of HCF (0, 1, 3, 5 and 10%) at day 0 (D0); Follow up by data collection of the tissue surface area from day 1 (D1) until day 10 (D10) and contraction analysis carried at day 5 (D5) and day 10 (D10). hPSC = human embryonic stem cell; CMs = Cardiomyocytes; MG = Matrigel; MM = Maturation medium; SF = Serum free; HCF = Human adult cardiac fibroblast. Created with BioRender.com.

The experiments were done using a double fluorescent reporter hPSC line (NKX2.5^EGFP/+-^COUP-TFII^mCherry/+^ generated in the human embryonic stem cell line HES3) [21]. HPSCs were maintained as undifferentiated colonies in Essential 8 medium (Thermo Fisher, A1517001) on vitronectin (Thermo Fisher, A31804)-coated 6-well plates. The differentiation to hPSC-CMs was induced as described previously [22]. Briefly, one day before starting the differentiation, hPSC were seeded at a density of 20-25x10^3^ cells per cm^2^ on Matrigel (83 μg protein/mL, Corning, 354230) coated 6-well plates in Essential 8 medium. After 24 h (D0), mesodermal differentiation was induced by addition of Activin-A (20–30 ng/mL, Miltenyi 130–115–010), BMP4 (20–30 ng/mL, R&D systems 314-BP/CF) and Wnt activator CHIR99021 (1.5–2.25 μmol/L, Axon Medchem 1386) in BPEL medium [23]. At day 3, cells were refreshed with BPEL containing WNT inhibitor XAV939 (5 μmol/L, R&D Systems 3748) and Matrigel (41.3 μg protein/mL). Cells were refreshed with BPEL on day 7 and 10 of differentiation. Beating CMs at day 13 were metabolically selected with a lactate purification step of 4 days. This lactate purification medium consisted of our previously described maturation medium (MM) [22] without glucose and with additional 5 mM of sodium DL-lactate solution (60%, Sigma Aldrich, cat. no. L4263). At day 17, purified CMs were kept in the above described lactate purification medium with additional 4.5 mM of glucose for three more days, when cells were dissociated with TrypLE 10X (ThermoFisher, A1217702) and cryopreserved (Fig 1A). Cells with at least 90% of green fluorescent protein (GFP) positive signal were used (S1 Fig).

### Fibroblast culture

The Human adult cardiac fibroblast (HCF) were obtained from Promocell (C-12375) and they were expanded according to the protocol [24]. Briefly, a T175 cell culture flask (Greiner) was incubated (at 37°C and 5% CO_2_) with 12 mL of FGM-3 (Promocell, C-23130) for 30 minutes. The cryovial containing the HCF was thawed in a water bath at 37°C. Then, the cells were transferred from the cryovial to a cell culture flask containing the FGM-3 with an additional 18 ml of FGM-3. Subsequently, the culture flask was placed inside the incubator. Refreshments were done every 48 hours. When the cells reached 70–90% confluency they were passaged to a new flask; this process was repeated until reaching 11 passages. At the last passage, the HCF were frozen at a final concentration of 150 x 10^3^cells/ 0.5 mL in freezing medium. The freezing medium consists of 50% KOSR (Thermo Fisher, 10828028), 40% FGM-3, 10% DMSO (Sigma-Aldrich, D2650) and 0.5% Revitacell (Thermo Fisher, A2644501).

### EHT formation

Cardiac EHTs were made as previously described from Ribeiro et al [25]. Briefly, three tissues were made per well in a 12 well plate format with five different ratios of HCF: 0%, 1%, 3%, 5% and 10%. First, both cell types (CMs and HCF) were thawed and resuspended in MM with 4.5 mM glucose and 5 mM sodium DL-lactate. After counting, both cell types were mixed by adding different percentages of HCF (0%, 1%, 3%, 5% or 10%) to a fixed amount of CMs (8x10^5^ cells for one well of the 12 well plate). Immediately after each group was centrifugated and the pellet was resuspended to a final concentration of 16.3x10^6^ cells/mL, 16.5x10^6^ cells/mL, 16.8x10^6^ cells/mL, 17x10^6^ cells/mL and 18x10^6^ cells/mL for each group, respectively. Subsequently, cells were mixed with an extracellular matrix (ECM) mixture consisting of 2X MM medium, fibrinogen (final concentration 2 mg/mL, Sigma-Aldrich F8630), Matrigel (final concentration 1 mg/mL) and aprotinin (final concentration 2.5 μg/mL, Sigma-Aldrich, A1153). Then, 0.6 U/mL of thrombin (Sigma, T7513) was added to the mix and quickly after mixing, 15 μl was used to make each one of the three tissues per well [25]. After 24 hours, the first refreshment was done and after that, the refreshments were done every 2 or 3 days and after contraction measurements.

### Data collection

EHTs in the 12 well plate were maintained at 37°C in 5% CO_2_ during image analysis. To assess EHT compaction over time, images of each tissue were taken after 24 hours of tissue formation for 10 days for automatic analysis of the surface area using our software (see below). Force of contraction was measured after 5 (D5) and 10 (D10) days of the tissues being formed by using a custom-made pacing device at 1 Hz (10 ms biphasic pulses, 4–5 V/cm) for 10 seconds. For all measurements, we used a Nikon Ti2-E inverted microscope with a high-speed camera Prime BSI from Photometrics at 100 fps with 2X magnification.

### Software tool description

*EHT Analysis* is an easy-to-use software tool that analyzes the contractile properties and contraction kinetics of EHTs. By tracking the center of the anchor points (pillars) where the tissue anchored to, *EHT Analysis* software extracts the displacement of the pillars as a consequence of tissue contraction, which is then converted into force (S2 Fig). From this displacement, the software calculates the maximum contraction and relaxation, as well as the contraction kinetics that includes the time that takes to achieve 10% and 90% of contraction and relaxation. All these parameters are important for assessing the patho-physiological properties of the tissues in normal or diseased conditions or following treatment of drug compounds. The software is run using parallel computing to decrease the time of the analysis.

**User interface (UI),** The current UI design has the following elements: an automatic and a manual mode. The automatic mode facilitates the analysis of a large amount of data automatically with just two clicks. If the user is particularly interested in one file and prefers either to select a specific region from the contraction waveform or to exclude a specific contraction pattern for further analysis, the manual mode gives that versatility.

**Fig 2:**
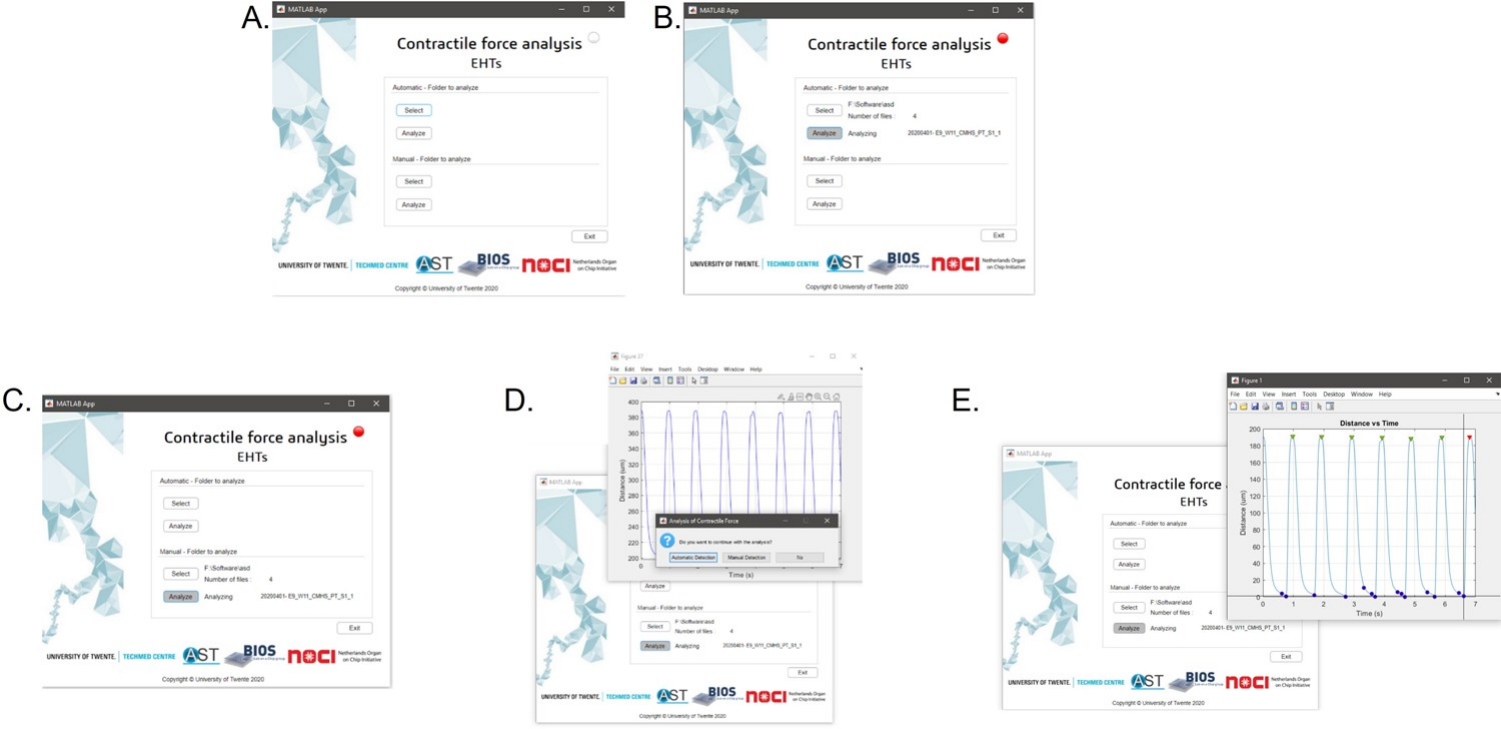
User interface *EHT analysis*. (A) Overview of the software. (B) Automatic contraction analysis option. (C-E) Manual contraction analysis per folder overview(C), selection of the analysis mode on preview contraction wave(automatic or manual) (D) and manually selection of maximums and minimums (E).

The automatic mode has two buttons, the first one allows to select the folder containing the files that needs to be analyzed and it will automatically display the name of the folder selected and the total number of files to be analyzed (Fig 2A). The second one is a “start” button that will start the analysis of each file automatically, while displaying the name of the file analyzing and as a visual confirmation the indicator will turn red during the analysis (Fig 2B). The manual mode (supervised) has the same two buttons but in this case, during the analysis a pop-up window with the preview graph of the displacement overtime there is the option to manually select the maximums and minimums or continue with the automatic analysis (Fig 2C–2E). When all the analyses are done the indicator will turn green as a visual sign. Additionally, there is an “exit” button if you want to close the UI at any moment.

**Fig 3:**
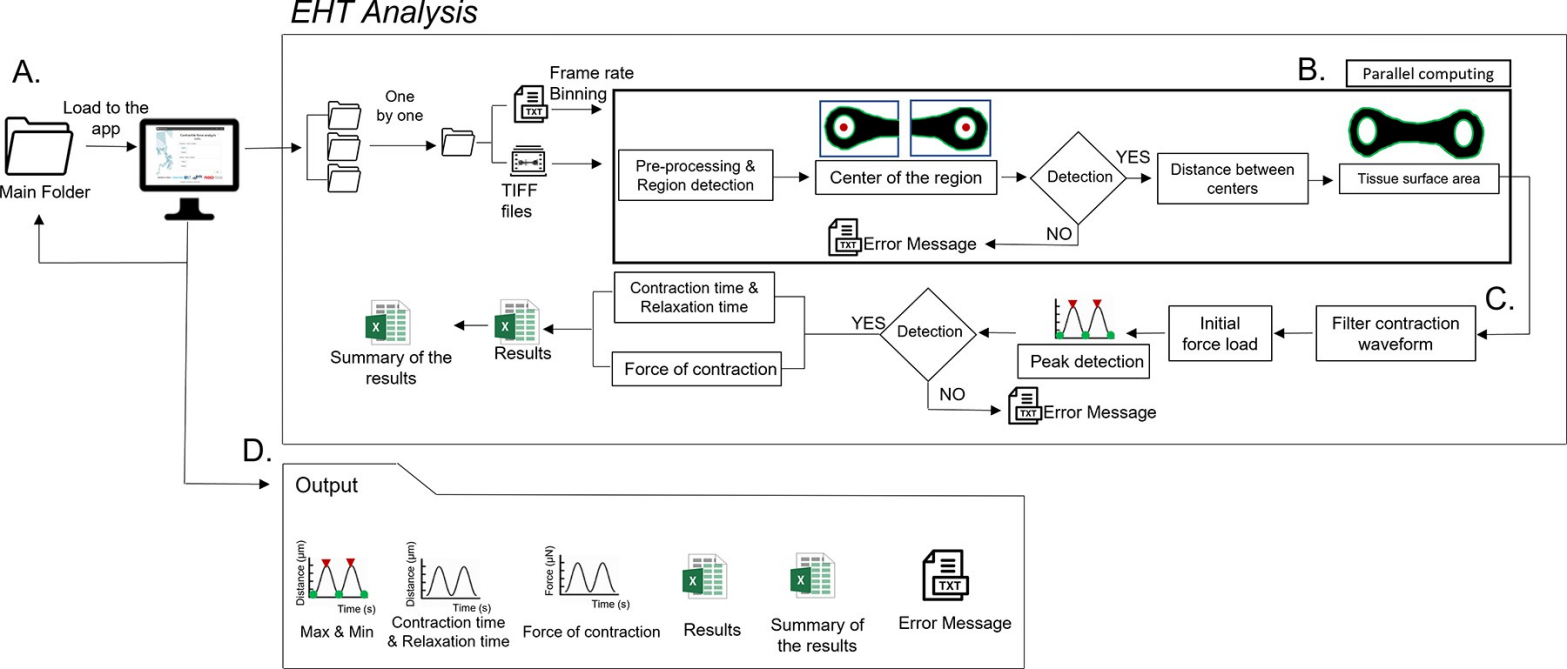
Flow chart representing the workflow of *EHT analysis*. (A) Automatic analysis of the required input data inside of a main folder. (B) Tracking the center of the pillars and the surface area, using parallel computing. (C) Contraction waveform post-processing. (D) Output data.

*EHT Analysis* workflow starts by choosing the main folder with the multiple files to be analyzed. Then, each file is analyzed sequentially. Inside of each file there is the bright-field tiff stack of images and a txt file with metadata of the microscope settings. From the metadata, the frame rate and the binning settings are extracted to calculate time and the size of a pixel to micrometers (μm) (Fig 3A). Segmentation is started on the tiff stack, by having each tiff picture divided in half to detect the center of the pillars. Furthermore, a pre-processing step is done to enhance the quality of the images. Regions that have artifacts like debris or dead cells on top of the pillars, that appear as black spots, are filled in with white pixels by taking into account pixel connectivity of 8 pixels. The image is then segmented into two levels by a specific image threshold that is defined using Otsu’s method [26] and converted to grayscale. The center of the pillars is determined by using the maximally stable external regions (MSER) algorithm to find the ellipses and centroids that fit into the regions. The MSER regions with low eccentricity (circular) are selected and from those the one with the biggest area is selected. The same process is done in the other half of the picture. Then, the distance between the centroids of the ellipses is calculated creating the contraction waveform and if a centroid is not found an error message is automatically generated. Later, the surface area of the tissue is calculated by extracting the region of the tissue. This is done by segmenting the whole image into two levels by a defined threshold (Otsu’s method [26]) and converting it into black and white image. To extract the region of the tissue, the color of the image is inverted, making the region of the tissue white and the background black. Then, the region of the pillars is filled with white pixels to have a complete segmented tissue. The tissue area in pixels is calculated by adding all the pixels inside the region that are found using the function regionprops and then converted to μm^2^. The area of the pillars (0.4 mm^2^) is subtracted from the total area in order to obtain just the area of the tissue. All these steps are done using the parallel computing toolbox of Matlab (Fig 3B).

The contraction waveform is smoothened using a Savitzky-Golay digital filter to eliminate any undesired noise before finding the maximums and minimums. The maximum threshold is used to find the peaks, it is defined as 20% from the top and the minimum threshold as 15% from the baseline. If there are no peaks detected, an error message is generated. Contraction kinetics are calculated as the time that takes to achieve 10% and 90% of contraction and relaxation. The contractile force (F [N]) is calculated using the displacement of the centroids over time (δ [s]), the length (L [m]) radius(R [m]), Young’s modulus(E [Pa]) and the position of the tissue (*a*[*m*]) on the pillar; accordingly to the elastic beam bending equation [27] (Fig 3C).


$$F=\frac{3\pi ER^{4}}{2a^{2}(3L-a)}\delta$$

All the information is saved into an excel file and four graphs are plotted (maximum & minimum displacement, contraction kinetics, contraction force and contraction force per surface area) per file. At the end of the analysis, an excel file with the average values of all the data is saved as a summary of the results and if there were errors, a txt file with the names of the files is generated (Fig 3D).

### Statistics

Statistical analysis was performed on GraphPad Prism 8. Differences between the different groups were assessed by two-way ANOVA plus Tukey’s post-Hoc test. Results are displayed as means ± s.e.m unless stated otherwise. Significance was attributed to comparisons with values of P <0.05 * P <0.01**; P <0.001***; P <0.0001****.

## Results

### Tracking algorithm

**Fig 4:**
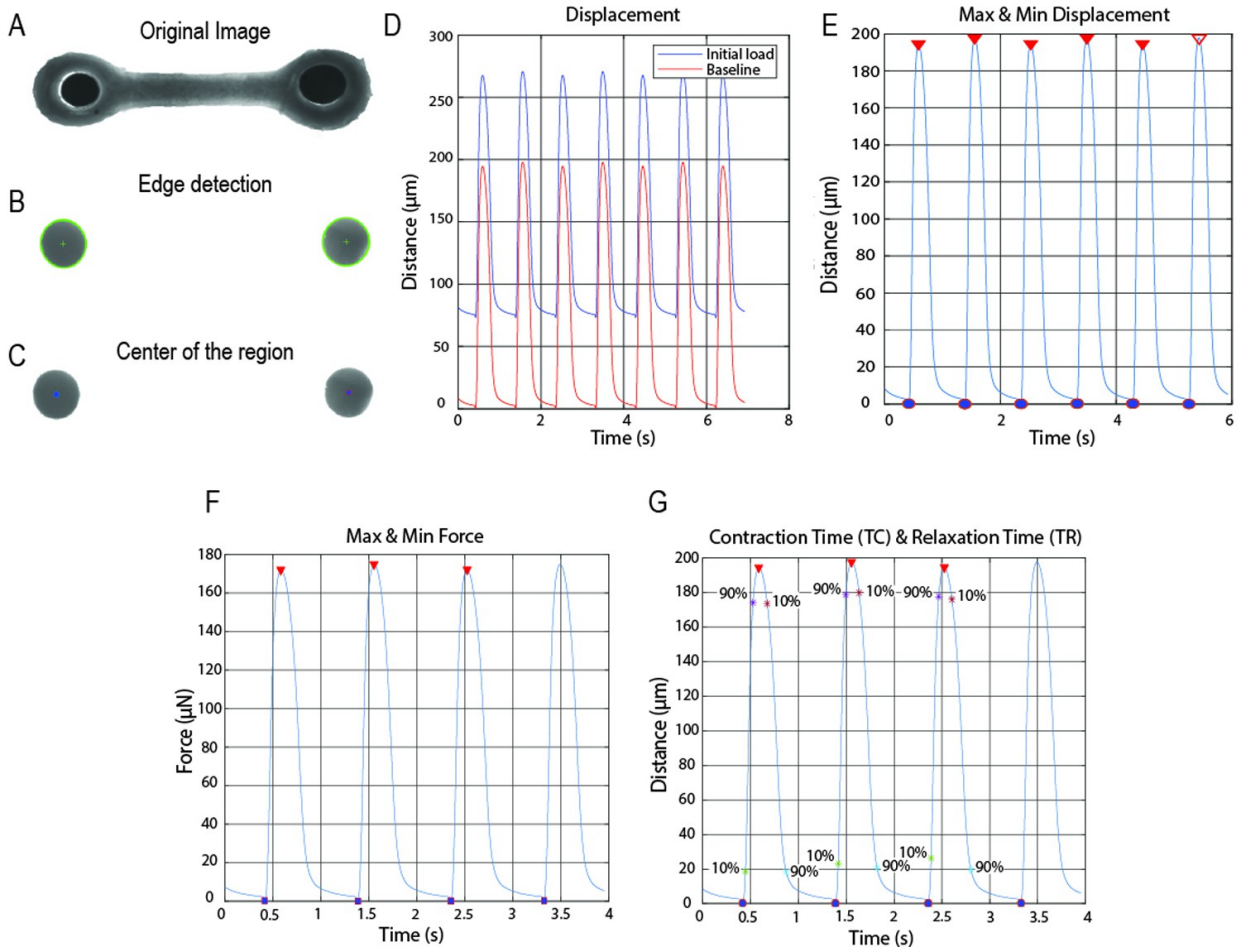
Contraction analysis by tracking black dots. (A) Original bright field image of an EHT around pillars with a black tip. (B) Edge detection of the black tip of the pillars from a bright field image. (C) Detection of the center of the pillars black region. (D) Displacement of the pillars over time with resting tension (Blue) and after removing the initial resting tension (Red). (E) Maximum and minimum detection of the contraction motion; complete contraction cycles are with filled marks. (F) Graph of the contraction force with the maximum and minimum. (G) Graph of displacement over time and also indication the moments to achieve 10% and 90% of contraction and relaxation.

Initially, to highlight the pillars tip location and simplify the tracking technic, the transparent PDMS tip of the pillars were painted using carbon black [25]. The carbon black allows to achieve a high level of blackness and reduces the light that passes through the material, resulting in clear black circles in the image (Fig 4A). At the moment of recording the light was increased until the point only the black dots of the pillars were visible, to increase the contrast with the background and facilitate the tracking of the pillars (Fig 4B). The two black regions were successfully detected and the center of each region was found using edge detection in each tiff image of the stack (Fig 4B and 4C). The displacement of the pillars was calculated by measuring the distance the center of the pillars displaced upon tissue contraction. The resting tension of the EHTs, upon tissue relaxation, was calculated as the difference of pillar position between the unloaded pillar (no tissue attached) and the loaded pillar (with tissue attached) [28] (Fig 4D, blue wave). After removing the resting tension, the algorithm identified correctly the peaks and periodicity of the contraction wave in an automated manner (Fig 4D, red wave). However, not all the videos are recorded at the starting point of a contraction cycle or ended at the end of it. Therefore, to calculate the force of contraction, contraction kinetics and the contraction time, a robust selection of the contractile motion was implemented to eliminate the calculation of incomplete contraction cycles (Fig 4E). The force of contraction was calculated taken into account the displacement of the pillars of the selected contraction cycles and the elastic beam bending equation [27] (Fig 4F). Then, from those cycles, the 10% and 90% of contraction and relaxation time and the contraction kinetics were calculated (Fig 4G). Furthermore, by using parallel computing, the time required to process 700 frames at 1320 x 477 pixel resolution decreases from 4 minutes on average to a maximum of 1.5 minutes. Therefore, the computational time for analyzing one tiff stack was significantly decreased depending on the number of available computing cores.

### Removal of black dots to capture automated tissue imaging

**Fig 5:**
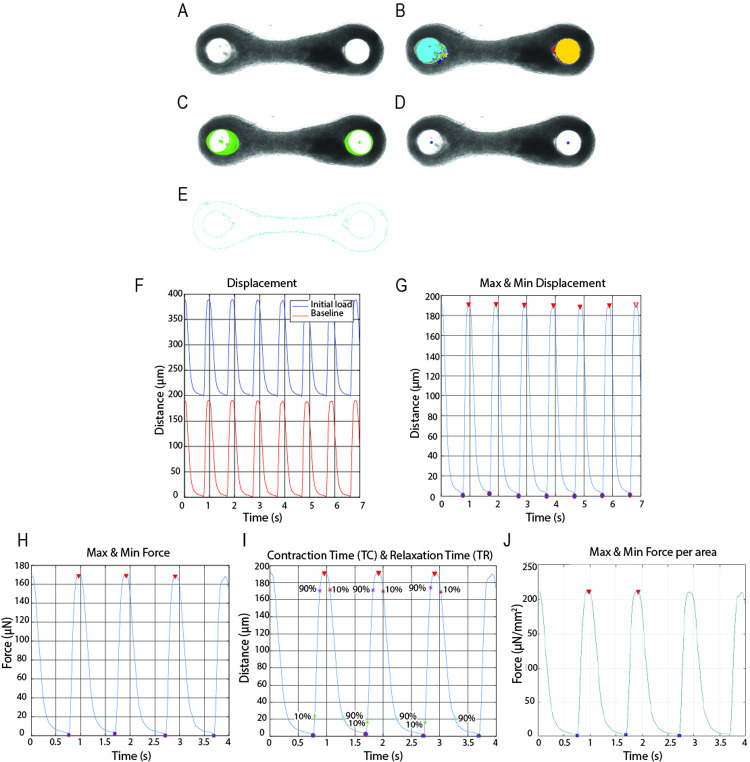
EHT contraction analysis on transparent pillars. (A) Original bright field image of an EHT around transparent pillars. (B) Region detection of the tip of the pillars from a bright field image. (C) Detection of the regions with low eccentricity. (D) Detection of the center of the pillars. (E) Contour of the EHT. (F) Displacement of the pillars over time with resting tension (Blue) and after removing the initial resting tension (Red). (G) Maximum and minimum detection of the contraction motion; complete contraction cycles are with filled marks. (H) Graph of the contraction force with the maximum and minimum.(I) Graph of displacement over time with the time to achieve 10% and 90% of contraction and relaxation. (J) Graph of force per surface area with maximum and minimum.

It has been shown that the extracellular matrix (ECM) remodeling and compaction of tissues are relevant for achieving a higher contractile performance [29, 30]. Thus, to include the formation of the tissues in the automatic analysis, we modified the fabrication of the pillars by eliminating the black paint on the tip and lowering the light intensity in order to record the tissues in the bright field image (Fig 5A). The tracking algorithm was successfully adapted to identify the region of the pillars (Fig 5B), select the ones with low eccentricity (circular), biggest area and detect the center of the pillars (Fig 5C, 5D and S2 Fig). Furthermore, the contour of the tissue was accurately extracted from the bright field and the surface area was calculated (Fig 5E). Using the same logic of the previously mentioned algorithm, the graphs of displacement, contraction force and time of contraction over time were generated accordingly (Fig 5F–5I). Additionally, using the tissue surface area calculated from the segmented tissue, force per surface area was plotted (Fig 5J).

### Contractile performance of EHT using different ratios of human adult cardiac fibroblast

**Fig 6:**
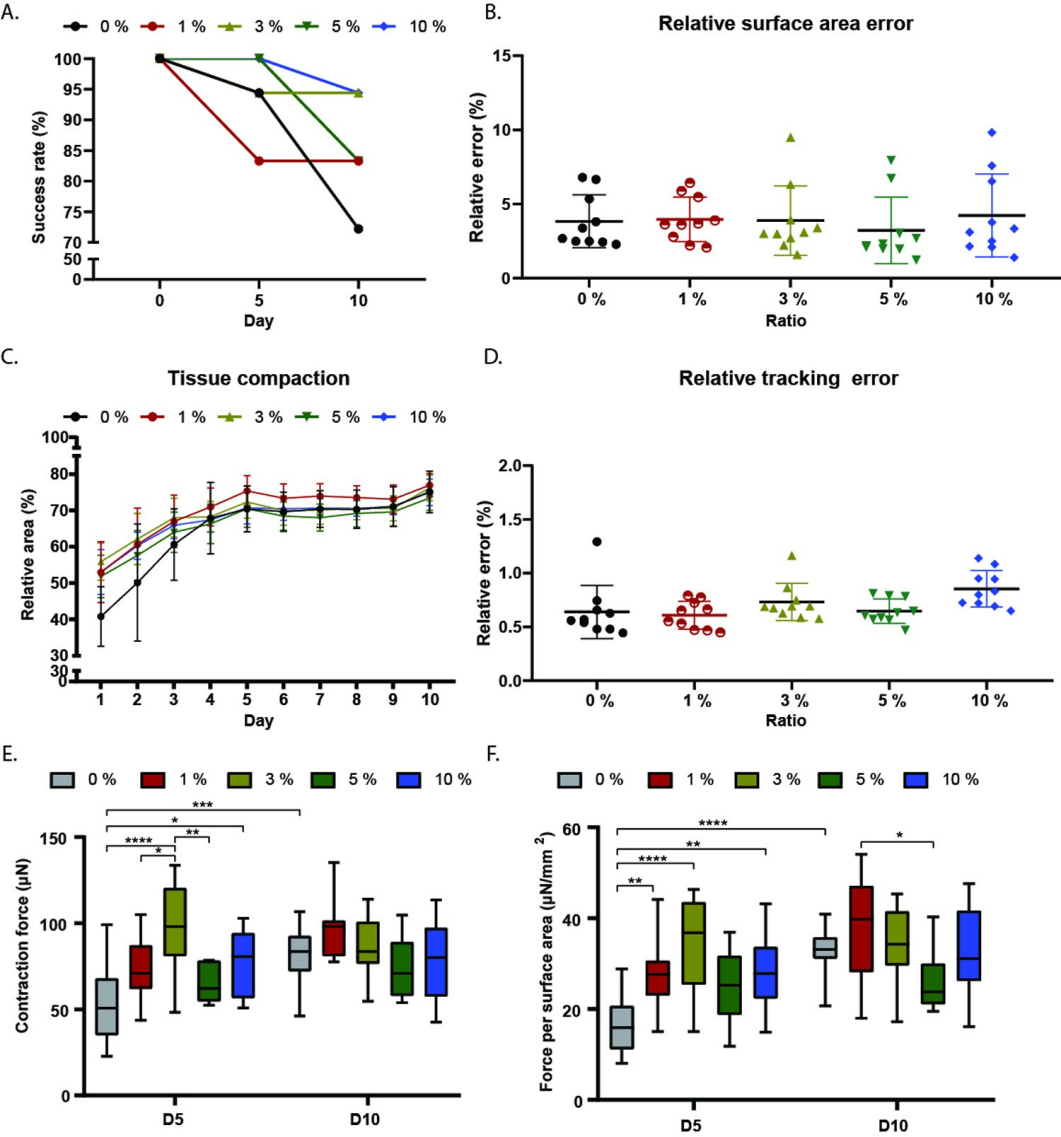
EHT comparison with different ratio of Human adult cardiac fibroblast (HCF). (A) Success rate of tissue formation using different ratio of HCF. (B) Relative surface area error by comparing the tissue area measured manually using Image J and using the stand alone application. (C) Relative tissue compaction by comparing the initial tissue area at day 0 (10.7 mm^2^) with the tissue area over time with the different ratio of HCF. (D) Relative tracking error of the pillar’s center by comparing the distance measured manually using Image J and using the stand alone application. (E) Contractile force of EHTs with different ratios of HCF at day 5 and 10. (F) Contractile force of EHTs with different ratios of HCF divided by the surface are of the tissue at day 5 and 10. In E-F, data shown as means, maxima and minima; Two-way ANOVA plus Tukey’s test for comparisons among ratios of HCF; * = p <0.05; ** = p <0.01; *** = p <0.001; **** = p <0.0001,(N = 3).

There is a great interest to define the best ratio of human adult cardiac fibroblast (HCF) on EHTs that enhances the contractile output in order to resemble *in vivo* situation. Therefore, we have applied this image analysis software to study this biological question. We successfully made EHTs from the hPSC line (NKX2.5^EGFP/+-^COUP-TFII^mCherry/+^) with different ratios of HCF (0%, 1%, 2%, 3%, 5% and 10%), by using our previously developed platform [25]. First, we evaluated the success rate of tissue formation over time, which is the homogenous formation of the 3D cardiac tissue around the pillars without any gap of cells in the middle (S3 Fig). The highest success rate of tissue formation was achieved by using 3% (94.4%, 17 of 18 tissues) and 10% (94.4%, 17 of 18 tissues) of HCF, followed by 1% (83.3%, 15 of 18 tissues), 5% (83.3%, 15 of 18 tissues) and 0% (72.2%, 13 of 18 tissues) (Fig 6A).

HCFs have shown to contribute to remodeling of the ECM and increase tissue compaction [29]. Before assessing the tissue compaction over time, we first evaluated the accuracy of the algorithm to segment and calculate the tissue area from the bright field images by comparing with the tissue area measured manually using the image processing software package Image J. During 10 days, on every day an image was taken and analyzed with both methods. In all calculated and measured tissue areas, the area of the pillars was subtracted. We found that the relative error over the 10 days of tissue area measurements using the automatic algorithm, was on average below 5% in all the conditions (Fig 6B). In terms of tissue compaction over time, the first 5 days were the ones where the biggest change in compaction was observed in all the cases. This is followed by a plateau phase after day 5 until day 10. Note that 1% and 3% of HCF showed the highest compaction changes during the initial 5 days and in general 1% of HCF showed the highest levels of tissue compaction over time (Fig 6C).

Next, we evaluated the accuracy of the algorithm to identify the center of the pillars from the bright field images by comparing with the distance measured manually using Image J. We used the same data set previously described for the relative surface area error. We found that the relative error of tracking the center of the pillars using the automatic algorithm, was on average below 1% in all the conditions (Fig 6D). Then, contractile properties of the EHTs with the different HCF ratios were measured on day 5 and 10. At day 5, the EHTs with 3% HCF showed a significantly higher contraction force compared to the other conditions except for 10% HCF, whereas at day 10 no significant differences in contraction force were observed in any of the cases. (Fig 6E). Additionally, we compared the performance of *EHT Analysis* with the software MUSCLEMOTION by analyzing 5 different bright field videos randomly selected with both software (S4 Fig).

The absolute values of contraction force make it difficult to compare the results from different research groups. Different platforms are used to make the EHTs and the differences herein creates variability in the measured contraction force. To facilitate the comparison of contractile force, the physiological cross-section area (PCSA) has been used in multiple studies [31–33]. However, this limits the throughput of analyzing multiple 3D cardiac tissues in an unbiased, fast manner and without the handling human error. Normally, to quantify the PCSA of the tissues a histology process is required; which limits the continuous analysis of the tissue over time. Instead, we analyzed the axial stress by dividing the contraction force per surface area. We found that tissues with 3% HCF showed significantly higher force per surface area compared to 0% and 1% HCF and tissue with 10% HCF compared to 0% HCF at day 5. While, at day 10 tissues with 1% HCF which showed higher axial stress compared with 5% HCF (Fig 6F).

**Fig 7:**
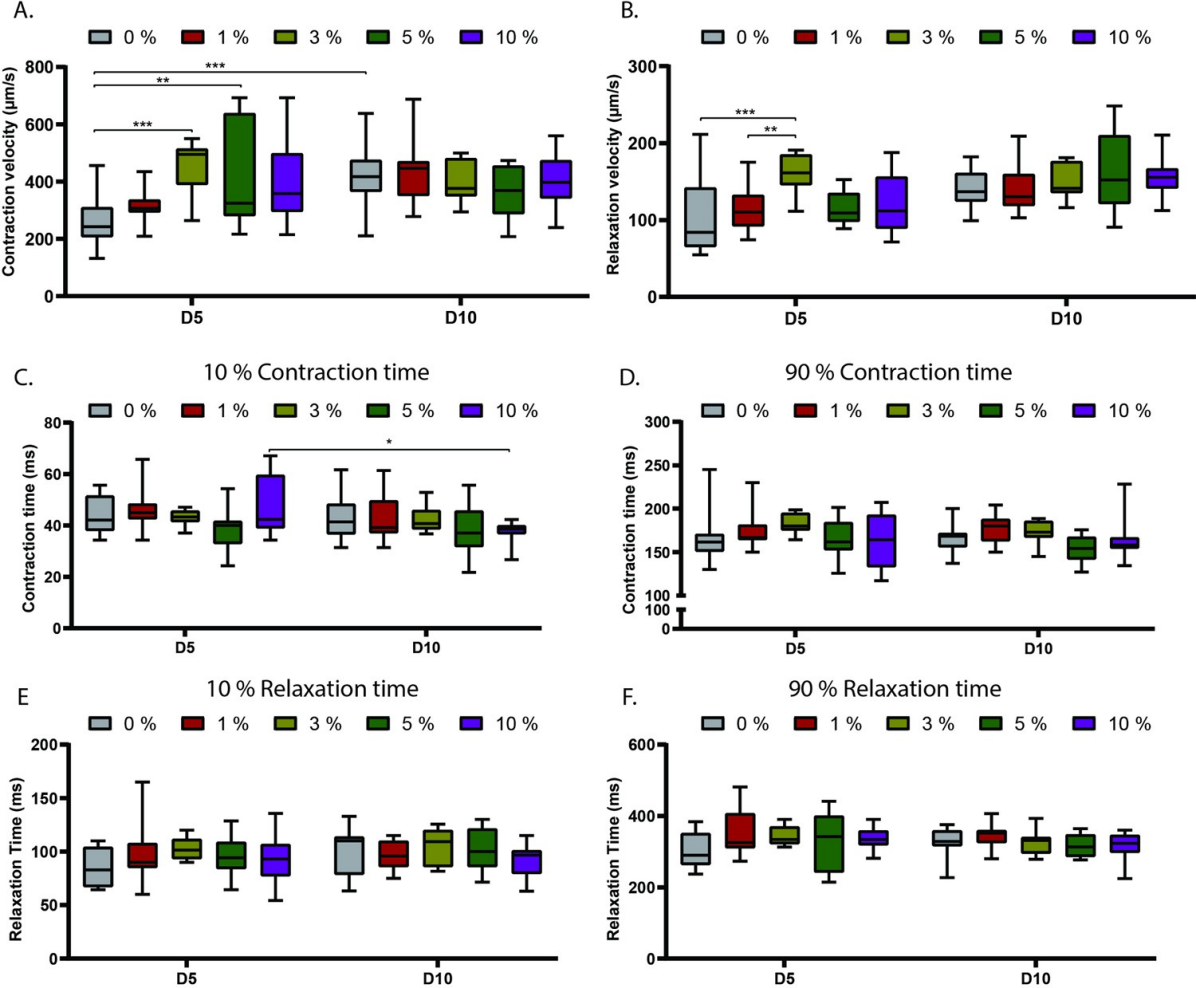
Contraction kinetics of EHTs with different ratio of human adult cardiac fibroblast (HCF). (A-B) Contraction (A) and relaxation (B) velocity of EHTs using different ratio of HCF at day 5 and 10. (C-D) Time to reach 10% (C) and 90% (D) of contraction using different ratio of HCF at day 5 (D5) and day 10 (D10). (E-F) Time to reach 10% (C) and 90% (D) of relaxation using different ratio of HCF at day 5 (D5) and day 10 (D10). Data shown as means, maxima and minima; Two-way ANOVA plus Tukey’s test for comparisons among ratios of HCF; * = p <0.05; ** = p <0.01; *** = p <0.001; **** = p <0.0001, (N = 3).

Other important contractile properties are the velocity of contraction and relaxation. Which are automatically calculated by the algorithm. EHTs with 3% and 5% of HCF displayed a significantly higher contraction velocity compared to EHTs with 0% HCF at day 5. While at the same timepoint, only EHTs with 3% HCF showed higher relaxation velocity compared to EHTs with 1% and 0% HCF. (Fig 7A and 7B). No significant differences were observed in the time that takes to achieve 10% and 90% of contraction and relaxation (Fig 7C–7F).

## Discussion

The increased use of 3D cardiac tissues with cylindrical pillars as anchor points as *in vitro* model for disease modeling and drug screening [27, 31, 34, 35], has opened a need for a standardized, efficient computing and reliable way to analyze physiological properties of the cardiac tissues.

In this study we have developed “*EHT analysis*”, a standalone application to analyze contractile properties of EHTs. This application with an easy-to-use interface has the advantage to automatically analyze multiple contraction motions of EHTs from high speed videos with low computation time in an unbiased way. Furthermore, the generated output of *EHT analysis*, facilitates the post-analysis by giving the results of all the contractile properties of each analyzed tissue in one excel file. This leads to an increase in productivity and a reduction of human errors during the analysis. Additionally, the user is able to access a detailed analysis per EHT with the data and graphs (displacement, contraction force, contraction kinetics and force per surface area over time), and in the specific case where the software cannot analyze the contraction motion of the tissue, a txt file will be generated automatically pointing out the name of the file, making it easy to find. Furthermore, it also has an option to do a supervised analysis for a more controlled or specific case analysis. Here, it offers the possibility to decide to analyze the contraction motion, making it a versatile tool for analyzing contractile properties of EHTs. The previous reported custom-made software developed by other researchers analyzed contraction force using different tracking techniques. In the case of the EHT Technologies platform [36], they focused on the top and bottom of the tissue strip by using a customized software package developed by a private company, which make it platform specific and not easy to access. While Serra et al [27], used a similar approach by tracking the centroids of the pillars, it is not clear how automated this analysis is and what variables besides contraction force and frequency are obtained as an output, which limits its use. Alternatively, fluorescent pillars have been used to track their position [37]. However, the accuracy of this method depends on the correct labeling of the pillars and the comparison with the theoretical grid of undeflected pillars. Any subtle variation in the initial distance of the pillars will introduce error in the measurement. In addition, MUSCLEMOTION [38] uses the differences in pixel intensity between a reference frame and the frame of interest for the assessment of the contraction. Although this an effective approach, assessment is sensitive to background noise and the automated selection of the frame of reference. High background levels or the wrong selection of the reference frame, affect the performance of the algorithm and introduce incorrect values as we observed (S4A Fig). Therefore, analysis using MUSCLEMOTION usually require large input by the user or training to analyze the files and may yield variability in output data. Consequently, contractile analysis is time-consuming, which limits the analysis throughput and comparison of data.

Previously reported force per PCSA has been used to compare the contractility performance of 3D cardiac tissues among research groups and different EHTs platforms [39–44]. However, force per PCSA is a factor that reduced the throughput of analysis and is time-consuming for a big scale experiment due to the histology step that is required to measure the PCSA of the tissues. With the *EHT Analysis* app we propose to analyse the axial stress, which is the force per surface area. This allows having a readout over time of tissue compaction and axial stress, without limiting analysis to an endpoint. In this way the PCSA of the tissue could be measured and included in the analysis after the histology step is done.

Additionally, we next used the *EHT Analysis* app to analyse the changes over time of tissue compaction and contractile properties of EHTs from hPSC-CMs with different ratios (0%, 1%, 3%, 5% and 10%) of HCF. We observed changes in the tissue compaction in the EHTs with HCF during the first 5 days of culture, while tissues without HCF took longer to compact. These findings correspond to previously reported influence of fibroblast in the remodeling kinetics of the ECM [45, 46]. Overall, the tissues with 3% HCF showed high success rate of tissue formation (94.4%), a highest level of tissue compaction and the higher contractile force and contraction kinetics in just 5 days of culture, which is promising for short experiments. Moreover, using 3% HCF is an alternative to move towards co-culture of different cell types in EHTs with a high success of tissue formation.

We demonstrated the versatility and advantage of using the *EHT analysis* standalone application to analyse hallmark physiological features of EHTs in an automatic, robust, unbiased and with low computational time of high speed bright field videos of EHT contraction motion. This makes it very useful and relevant for the analysis of big data sets result of high-throughput experiments of drug testing and disease modelling. Furthermore, the automatic surface area measurement is a valuable readout that can facilitate investigations like the effect of cardiac fibrosis [47]. For a next step, we believe that the use of an open software platform will be required to make the application more accessible.

## Conclusion

In this study, we developed a standalone application “EHT Analysis” to automatically analyze hallmark physiological features of EHTs, formed around cylindrical anchor points, in a standardized, robust, unbiased and with low computational time. This app will be useful to increase the speed of analysis and reduce human error in measurements, which will be beneficial for drug discovery and disease modeling applications.

## Supporting information

S1 Fig Cardiomyocyte differentiation. Representative histogram plot of flow cytometry of differentiated COUP-red (NKX2.5^eGFP/+^-COUP-TFII^mCherry/+^) CMs after lactate purification at day 20. Cardiomyocytes are quantified with the percentage of NKX2.5^eGFP+)^ positive cells. Grey: negative control (NKX2.55^Egfp-)^ negative cells), green: NKX2.5^eGFP+)^ positive cells.
(TIF)

S2 Fig EHT video. Example of tracking the center of the pillars of a EHT bright field video, record with a 2x magnification.
(MP4)

S3 Fig Tissue compaction over time. EHTs with different ratios of human adult cardiac fibroblast at day 0 (D0),3 (D3), 5 (D5) and 10 (D10).
(TIF)

S4 Fig Comparison between *EHT analysis* and MUSCLEMOTION. A-E Results of 5 different brightfield videos of EHT contraction using *EHT analysis* and MUSCLEMOTION.
(JPG)

## References

[1] Dunbar SB, Khavjou OA, Bakas T, Hunt G, Kirch RA, Leib AR, et al. Projected Costs of Informal Caregiving for Cardiovascular Disease: 2015 to 2035: A Policy Statement From the American Heart Association. Circulation 2018;137:e558–77. doi: 10.1161/CIR.0000000000000570 29632217

[2] Bansilal S, Castellano JM, Fuster V. Global burden of CVD: Focus on secondary prevention of cardiovascular disease. Int J Cardiol 2015;201:S1–7. doi: 10.1016/S0167-5273(15)31026-3 26747389

[3] Timmis A, Townsend N, Gale CP, Torbica A, Lettino M, Petersen SE, et al. European society of cardiology: Cardiovascular disease statistics 2019. Eur Heart J 2020;41:12–85. doi: 10.1093/eurheartj/ehz859 31820000

[4] Khavjou O, Phelps D, Leib A. Projections of Cardiovascular Disease Prevalence and Costs: 2015–2035. RTI Int 2016:1–54.

[5] Mittal R, Woo FW, Castro CS, Cohen MA, Karanxha J, Mittal J, et al. Organ-on-chip models: Implications in drug discovery and clinical applications. J Cell Physiol 2019;234:8352–80. doi: 10.1002/jcp.27729 30443904

[6] Lelièvre SA, Kwok T, Chittiboyina S. Architecture in 3D cell culture: An essential feature for in vitro toxicology. Toxicol Vitr 2017;45:287–95. doi: 10.1016/j.tiv.2017.03.012 28366709 PMC5623165

[7] Lemme M, Ulmer BM, Lemoine MD, Zech ATL, Flenner F, Ravens U, et al. Atrial-like Engineered Heart Tissue: An In Vitro Model of the Human Atrium. Stem Cell Reports 2018;11:1378–90. doi: 10.1016/j.stemcr.2018.10.008 30416051 PMC6294072

[8] Mannhardt I, Breckwoldt K, Letuffe-Brenière D, Schaaf S, Schulz H, Neuber C, et al. Human Engineered Heart Tissue: Analysis of Contractile Force. Stem Cell Reports 2016;7:29–42. doi: 10.1016/j.stemcr.2016.04.011 27211213 PMC4944531

[9] Sweeney HL, Hammers DW. Muscle contraction. Cold Spring Harb Perspect Biol 2018;10. doi: 10.1101/cshperspect.a023200 29419405 PMC5793755

[10] Abi-Gerges N, Miller PE, Ghetti A. Human Heart Cardiomyocytes in Drug Discovery and Research: New Opportunities in Translational Sciences. Curr Pharm Biotechnol 2019;21:787–806. 10.2174/1389201021666191210142023. 31820682

[11] Stein JM, Mummery CL, Bellin M. Engineered models of the human heart: directions and challenges. Stem Cell Reports 2020;0. doi: 10.1016/j.stemcr.2020.11.013 33338434 PMC8452488

[12] Mills RJ, Parker BL, Quaife-Ryan GA, James DE, Porrello ER, Hudson Correspondence JE. Drug Screening in Human PSC-Cardiac Organoids Identifies Pro-proliferative Compounds Acting via the Mevalonate Pathway. Cell Stem Cell 2019;24:895–907. doi: 10.1016/j.stem.2019.03.009 30930147

[13] Zhao Y, Rafatian N, Feric NT, Cox BJ, Aschar-Sobbi R, Wang EY, et al. A Platform for Generation of Chamber-Specific Cardiac Tissues and Disease Modeling. Cell 2019;176:913–927.e18. doi: 10.1016/j.cell.2018.11.042 30686581 PMC6456036

[14] Schaaf S, Shibamiya A, Mewe M, Eder A, Stö Hr A. Human Engineered Heart Tissue as a Versatile Tool in Basic Research and Preclinical Toxicology. PLoS One 2011;6:26397. doi: 10.1371/journal.pone.0026397 22028871 PMC3197640

[15] Feric NT, Pallotta I, Singh R, Bogdanowicz DR, Gustilo MM, Chaudhary KW, et al. Engineered Cardiac Tissues Generated in the Biowire II: A Platform for Human-Based Drug Discovery. Toxicol Sci 2019;172:89–97. 10.1093/toxsci/kfz168. PMC6813749 31385592

[16] Ulmer BM, Stoehr A, Schulze ML, Patel S, Gucek M, Mannhardt I, et al. Contractile Work Contributes to Maturation of Energy Metabolism in hiPSC-Derived Cardiomyocytes. Stem Cell Reports 2018;10:834–47. doi: 10.1016/j.stemcr.2018.01.039 29503093 PMC5919410

[17] Abilez OJ, Tzatzalos E, Yang H, Zhao MT, Jung G, Zöllner AM, et al. Passive Stretch Induces Structural and Functional Maturation of Engineered Heart Muscle as Predicted by Computational Modeling. Stem Cells 2018;36:265–77. doi: 10.1002/stem.2732 29086457 PMC5785460

[18] Ronaldson-Bouchard K, Ma SP, Yeager K, Chen T, Song LJ, Sirabella D, et al. Advanced maturation of human cardiac tissue grown from pluripotent stem cells. Nature 2018;556:239–43. doi: 10.1038/s41586-018-0016-3 29618819 PMC5895513

[19] Qu Y, Feric N, Pallotta I, Singh R, Sobbi R, Vargas HM. Inotropic assessment in engineered 3D cardiac tissues using human induced pluripotent stem cell-derived cardiomyocytes in the BiowireTM II platform. J Pharmacol Toxicol Methods 2020;105. doi: 10.1016/j.vascn.2020.106886 32629159

[20] Mannhardt I, Saleem U, Mosqueira D, Loos MF, Ulmer BM, Lemoine MD, et al. Comparison of 10 Control hPSC Lines for Drug Screening in an Engineered Heart Tissue Format. Stem Cell Reports 2020;15:983–98. doi: 10.1016/j.stemcr.2020.09.002 33053362 PMC7561618

[21] Schwach V, Verkerk AO, Mol M, Monshouwer-Kloots JJ, Devalla HD, Orlova V V., et al. A COUP-TFII Human Embryonic Stem Cell Reporter Line to Identify and Select Atrial Cardiomyocytes. Stem Cell Reports 2017;9:1765–79. doi: 10.1016/j.stemcr.2017.10.024 29173897 PMC5785710

[22] Birket MJ, Ribeiro MC, Kosmidis G, Ward D, Leitoguinho AR, van de Pol V, et al. Contractile Defect Caused by Mutation in MYBPC3 Revealed under Conditions Optimized for Human PSC-Cardiomyocyte Function. Cell Rep 2015;13:733–45. doi: 10.1016/j.celrep.2015.09.025 26489474 PMC4644234

[23] Ng ES, Davis R, Stanley EG, Elefanty AG. A protocol describing the use of a recombinant protein-based, animal product-free medium (APEL) for human embryonic stem cell differentiation as spin embryoid bodies. Nat Protoc 2008;3:768–76. doi: 10.1038/nprot.2008.42 18451785

[24] PromoCell. Instruction Manual. n.d.

[25] Ribeiro MC, Rivera-Arbeláez JM, Cofiño-Fabres C, Schwach V, Slaats RH, Den SA ten, et al. A New Versatile Platform for Assessment of Improved Cardiac Performance in Human-Engineered Heart Tissues. J Pers Med 2022, Vol 12, Page 214 2022;12:214. doi: 10.3390/jpm12020214 35207702 PMC8877418

[26] Otsu N. Threshold Selection Method from Gray-Level Histograms. IEEE Trans Syst Man Cybern 1979;SMC-9:62–6. 10.1109/TSMC.1979.4310076.

[27] Serrao GW, Turnbull IC, Ancukiewicz D, Kim DE, Kao E, Cashman TJ, et al. Myocyte-depleted engineered cardiac tissues support therapeutic potential of mesenchymal stem cells. Tissue Eng—Part A 2012;18:1322–33. doi: 10.1089/ten.TEA.2011.0278 22500611 PMC3397121

[28] Naito H, Melnychenko I, Didié M, Schneiderbanger K, Schubert P, Rosenkranz S, et al. Optimizing engineered heart tissue for therapeutic applications as surrogate heart muscle. Circulation 2006;114. doi: 10.1161/CIRCULATIONAHA.105.001560 16820649

[29] Fan D, Takawale A, Lee J, Kassiri Z. Cardiac fibroblasts, fibrosis and extracellular matrix remodeling in heart disease. Fibrogenesis Tissue Repair 2012;5:15. doi: 10.1186/1755-1536-5-15 22943504 PMC3464725

[30] Rodriguez ML, Graham BT, Pabon LM, Han SJ, Murry CE, Sniadecki NJ. Measuring the Contractile Forces of Human Induced Pluripotent Stem Cell-Derived Cardiomyocytes With Arrays of Microposts. J Biomech Eng 2014;136:0510051. doi: 10.1115/1.4027145 24615475 PMC4158804

[31] Leonard A, Bertero A, Powers JD, Beussman KM, Bhandari S, Regnier M, et al. Afterload promotes maturation of human induced pluripotent stem cell derived cardiomyocytes in engineered heart tissues. J Mol Cell Cardiol 2018;118:147–58. doi: 10.1016/j.yjmcc.2018.03.016 29604261 PMC5940558

[32] Sasaki D, Matsuura K, Seta H, Haraguchi Y, Okano T, Shimizu T. Contractile force measurement of human induced pluripotent stem cell-derived cardiac cell sheet-tissue. PLoS One 2018;13:e0198026. doi: 10.1371/journal.pone.0198026 29791489 PMC5965888

[33] Turnbull IC, Karakikes I, Serrao GW, Backeris P, Lee JJ, Xie C, et al. Advancing functional engineered cardiac tissues toward a preclinical model of human myocardium. FASEB J 2014;28:644–54. doi: 10.1096/fj.13-228007 24174427 PMC3898643

[34] Mannhardt I, Warncke C, Trieu HK, Müller J, Eschenhagen T. Piezo-bending actuators for isometric or auxotonic contraction analysis of engineered heart tissue. J Tissue Eng Regen Med 2019;13:3–11. doi: 10.1002/term.2755 30334614

[35] Tiburcy M, Hudson JE, Balfanz P, Schlick S, Meyer T, Liao MLC, et al. Defined engineered human myocardium with advanced maturation for applications in heart failure modeling and repair. Circulation 2017;135:1832–47. doi: 10.1161/CIRCULATIONAHA.116.024145 28167635 PMC5501412

[36] Hansen A, Eder A, Bönstrup M, Flato M, Mewe M, Schaaf S, et al. Development of a drug screening platform based on engineered heart tissue. Circ Res 2010;107:35–44. doi: 10.1161/CIRCRESAHA.109.211458 20448218

[37] Sniadecki NJ, Chen CS. Microfabricated Silicone Elastomeric Post Arrays for Measuring Traction Forces of Adherent Cells. Methods Cell Biol 2007;83:313–28. doi: 10.1016/S0091-679X(07)83013-5 17613314

[38] Sala L, van Meer BJ, Tertoolen LT, Bakkers J, Bellin M, Davis RP, et al. MUSCLEMOTION: A Versatile Open Software Tool to Quantify Cardiomyocyte and Cardiac Muscle Contraction In Vitro and In Vivo. Circ Res 2017:CIRCRESAHA.117.312067. doi: 10.1161/CIRCRESAHA.117.312067 29282212 PMC5805275

[39] Tiburcy M, Hudson JE, Balfanz P, Schlick S, Meyer T, Liao M-LC, et al. Defined Engineered Human Myocardium with Advanced Maturation for Applications in Heart Failure Modelling and Repair. Circulation 2017;135:1832. doi: 10.1161/CIRCULATIONAHA.116.024145 28167635 PMC5501412

[40] Sasaki D, Matsuura K, Seta H, Haraguchi Y, Okano T, Shimizu T. Contractile force measurement of human induced pluripotent stem cell-derived cardiac cell sheet-tissue. PLoS One 2018;13. doi: 10.1371/journal.pone.0198026 29791489 PMC5965888

[41] Ruan JL, Tulloch NL, Razumova MV, Saiget M, Muskheli V, Pabon L, et al. Mechanical Stress Conditioning and Electrical Stimulation Promote Contractility and Force Maturation of Induced Pluripotent Stem Cell-Derived Human Cardiac Tissue. Circulation 2016;134:1557–67. doi: 10.1161/CIRCULATIONAHA.114.014998 27737958 PMC5123912

[42] Masumoto H, Nakane T, Tinney JP, Yuan F, Ye F, Kowalski WJ, et al. The myocardial regenerative potential of three-dimensional engineered cardiac tissues composed of multiple human iPS cell-derived cardiovascular cell lineages. Sci Rep 2016;6. doi: 10.1038/srep29933 27435115 PMC4951692

[43] Tulloch NL, Muskheli V, Razumova MV, Korte FS, Regnier M, Hauch KD, et al. Growth of engineered human myocardium with mechanical loading and vascular coculture. Circ Res 2011;109:47–59. doi: 10.1161/CIRCRESAHA.110.237206 21597009 PMC3140796

[44] Goldfracht I, Protze S, Shiti A, Setter N, Gruber A, Shaheen N, et al. Generating ring-shaped engineered heart tissues from ventricular and atrial human pluripotent stem cell-derived cardiomyocytes. Nat Commun 2020 111 2020;11:1–15. doi: 10.1038/s41467-019-13868-x 31911598 PMC6946709

[45] Porter KE, Turner NA. Cardiac fibroblasts: at the heart of myocardial remodeling. Pharmacol Ther 2009;123:255–78. doi: 10.1016/j.pharmthera.2009.05.002 19460403

[46] Thavandiran N, Dubois N, Mikryukov A, Massé S, Beca B, Simmons CA, et al. Design and formulation of functional pluripotent stem cell-derived cardiac microtissues. Proc Natl Acad Sci U S A 2013;110. doi: 10.1073/pnas.1311120110 24255110 PMC3856835

[47] Wang EY, Rafatian N, Zhao Y, Lee A, Lai BFL, Lu RX, et al. Biowire Model of Interstitial and Focal Cardiac Fibrosis. ACS Cent Sci 2019;5:1146. doi: 10.1021/acscentsci.9b00052 31403068 PMC6661857

