# Supplementary figures and images for: Contractility analysis of human engineered 3D heart tissues by an automatic tracking technique using a standalone application
Source: PLoS One. 2022 Apr 14;17(4):e0266834. doi: 10.1371/journal.pone.0266834 (PMC9009597; doi:10.1371/journal.pone.0266834)

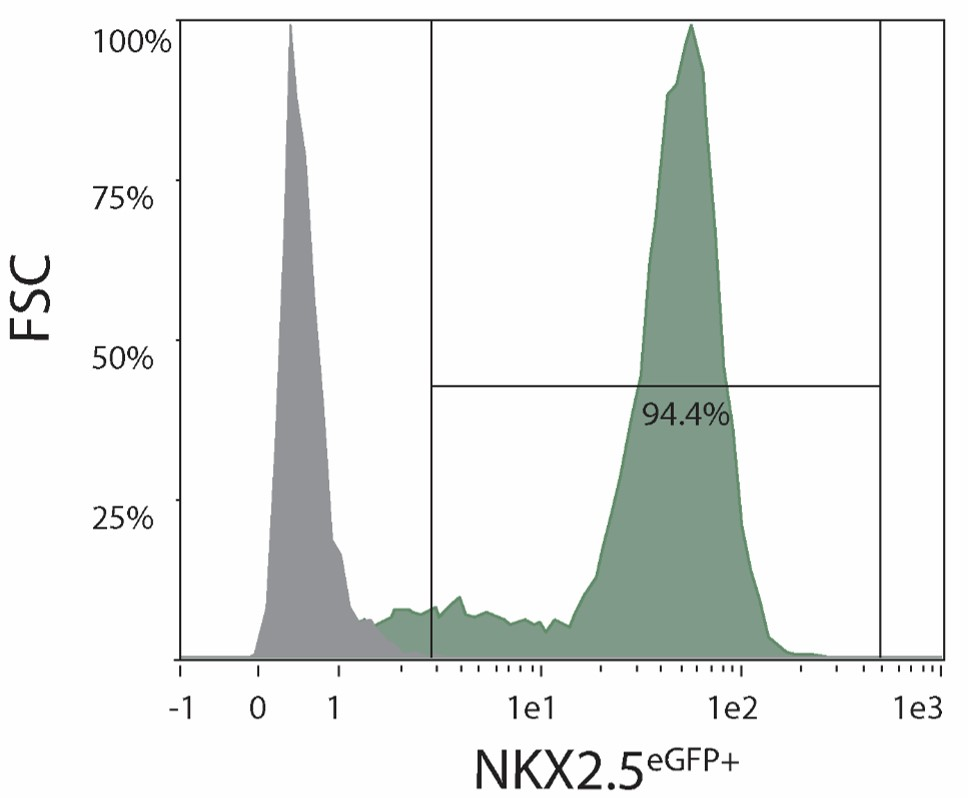

Supplement: S1 Fig — Representative histogram plot of flow cytometry of differentiated COUP-red (NKX2.5eGFP/+-COUP-TFIImCherry/+) CMs after lactate purification at day 20. Cardiomyocytes are quantified with the percentage of NKX2.5eGFP+) positive cells. Grey: negative control (NKX2.55Egfp-) negative cells), green: NKX2.5eGFP+) positive cells. (TIF) [file pone.0266834.s001.tif]

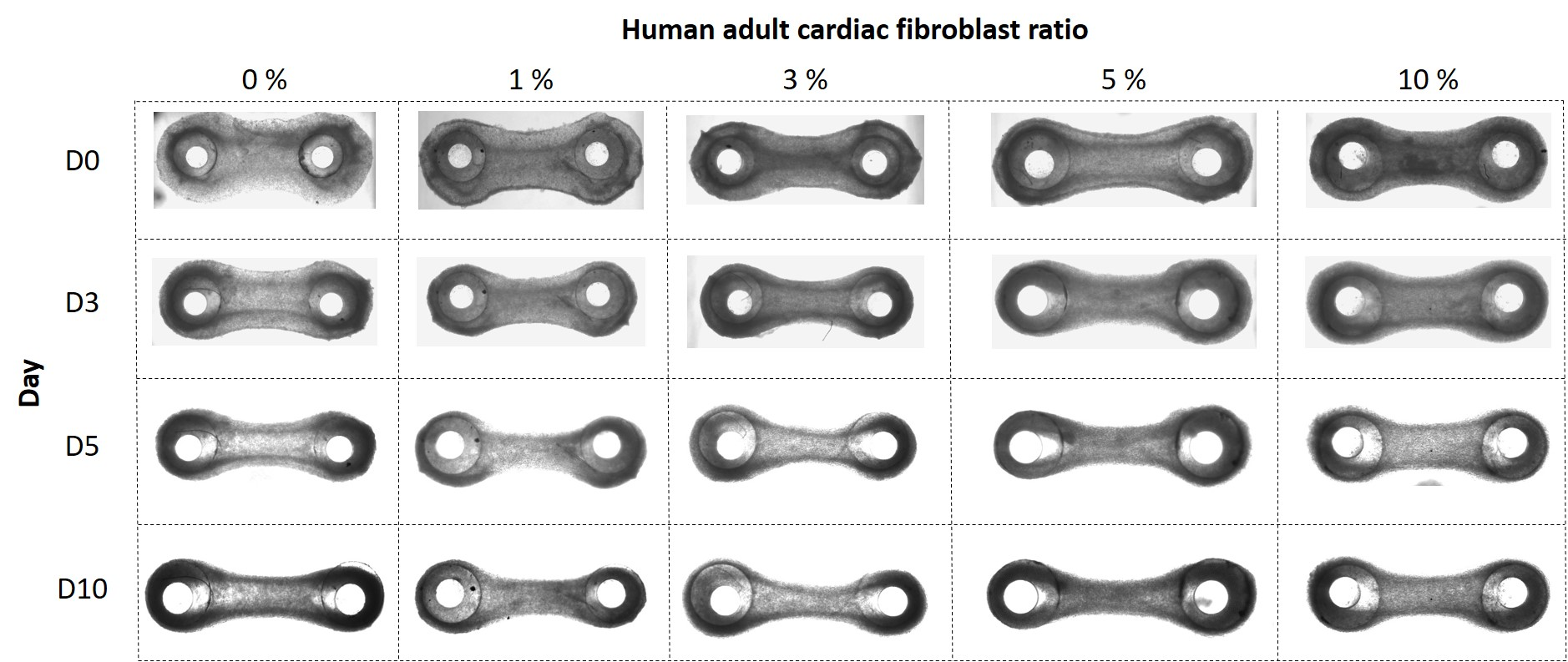

Supplement: S3 Fig — EHTs with different ratios of human adult cardiac fibroblast at day 0 (D0),3 (D3), 5 (D5) and 10 (D10). (TIF) [file pone.0266834.s003.tif]

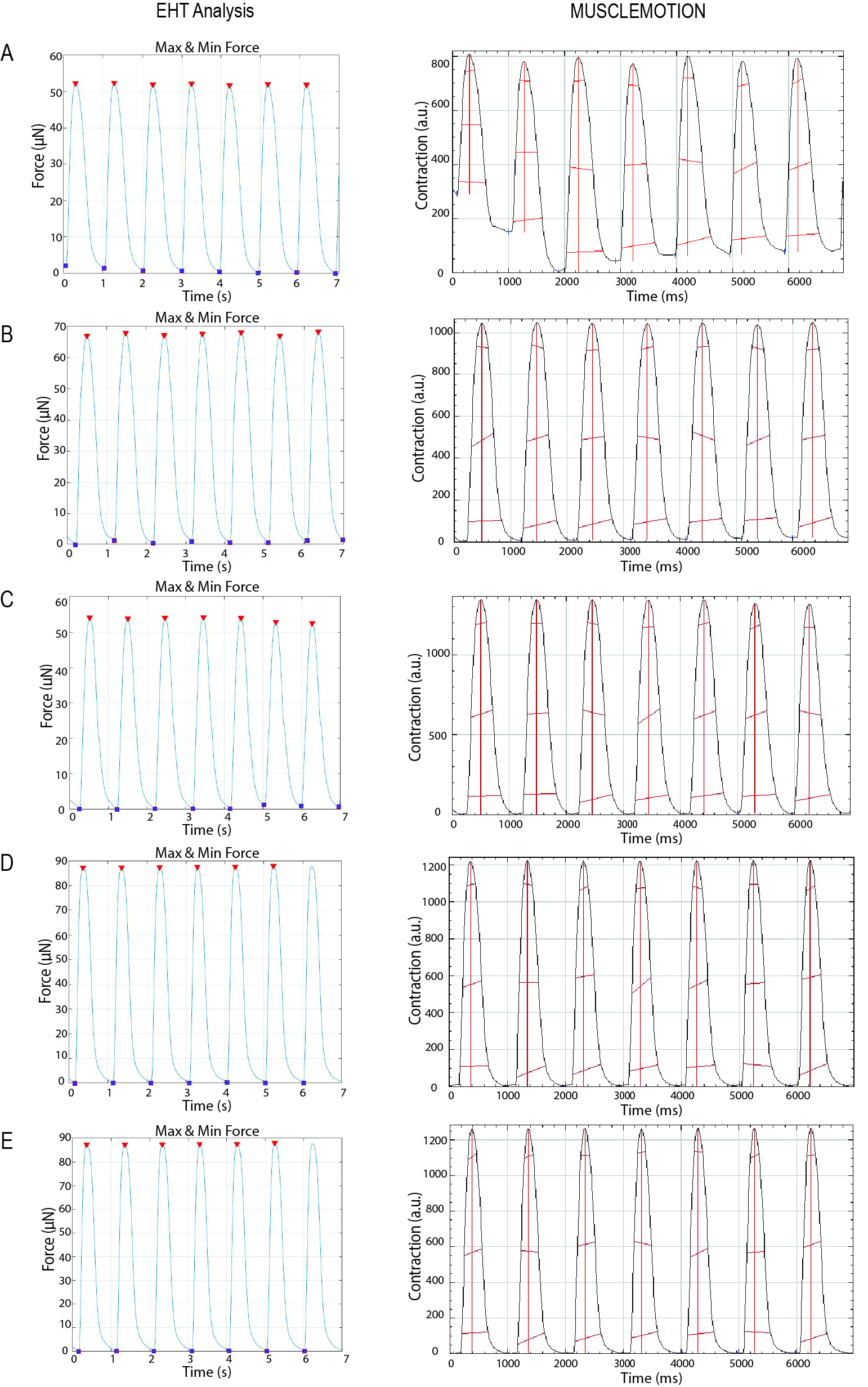

Supplement: S4 Fig — A-E Results of 5 different brightfield videos of EHT contraction using EHT analysis and MUSCLEMOTION. (JPG) [file pone.0266834.s004.jpg]
